# Clinical and Allelic Heterogeneity in a Small Cohort of Patients with Inherited Epidermolysis Bullosa

**DOI:** 10.3390/ijms26125762

**Published:** 2025-06-16

**Authors:** Anastasiia A. Buianova, Anastasia S. Yagizarova, Anastasiya V. Kosykh, Alexey A. Kubanov, Vera A. Belova, Anna O. Shmitko, Arfenya E. Karamova, Aleksandra A. Martynova, Grigoriy S. Podmoskovnikov, Maria A. Nefedova, Ekaterina S. Monchakovskaya, Dmitriy O. Korostin, Nadya G. Gurskaya, Denis V. Rebrikov

**Affiliations:** 1 Center for Precision Genome Editing and Genetic Technologies for Biomedicine, Pirogov Russian National Research Medical University, 1, bld. 1, Ostrovityanova St., Moscow 117513, Russia; anastasiiabuianova97@gmail.com (A.A.B.); anastasia.yagizarova@gmail.com (A.S.Y.); avkosyh@gmail.com (A.V.K.); verusik.belova@gmail.com (V.A.B.); annashmi97@gmail.com (A.O.S.); aleksandramartynova077@gmail.com (A.A.M.); podmoskovnikovr@yandex.ru (G.S.P.); d.korostin@gmail.com (D.O.K.); ncagip4@gmail.com (D.V.R.); 2 V.I. Kulakov National Medical Research Center for Obstetrics, Gynecology and Perinatology, Ministry of Healthcare, 4, Oparin St., Moscow 117198, Russia; 3 State Research Center of Dermatovenerology and Cosmetology, 3, bld. 6, Korolenko St., Moscow 107076, Russia; alex@cnikvi.ru (A.A.K.); karamova@cnikvi.ru (A.E.K.); nefedova.maria.arb@gmail.com (M.A.N.); monchakovskaya@cnikvi.ru (E.S.M.)

**Keywords:** epidermolysis bullosa, exome sequencing, pathogenic variant, premature termination codon, in silico, intron, splicing

## Abstract

Inherited epidermolysis bullosa (EB) comprises a group of genetic disorders characterized by fragile skin that blisters easily. Targeted therapies for EB necessitate personalized approaches, underscoring the importance of precise diagnostics through genetic analysis and skin biopsy using transmission electron microscopy and/or immunohistochemistry. This study highlights the application of whole-exome sequencing (WES) to identify key pathogenic variants associated with EB. Most identified variants were associated with the recessive form of dystrophic EB, including four novel *COL7A1* mutations: p.Leu1488ArgfsTer222, c.7759-3C>G, p.Gln1886Ter, and c.6501+6T>C, as well as recurrent variants p.Lys142Arg and p.Gly2049Glu. Additionally, variants were detected in *KRT5* (c.971T>C, p.Val324Ala), associated with EB simplex, and in *LAMB3* (c.2500C>T, p.Gln834Ter) in the homozygous state, associated with junctional EB. In silico splice prediction tools suggested disrupted splicing in both cases. One patient received topical gentamicin therapy targeting the nonsense mutation p.Gln1886Ter. These findings underscore the utility of WES in EB diagnostics, broaden the mutation spectrum, and contribute to the understanding of genotype–phenotype correlations in adult patients with EB.

## 1. Introduction

Inherited epidermolysis bullosa (EB) is a heterogeneous group of rare skin disorders characterized by mechanical fragility of affected tissues. The condition results from mutations in genes encoding structural proteins essential for maintaining cellular integrity and adhesion. This disruption compromises the dermal–epidermal junction, resulting in the development of blisters, erosions, and ulcerations on the skin and mucous membranes, particularly at sites of minimal mechanical trauma. Furthermore, structural and functional abnormalities in keratins may lead to keratinopathies, further compromising the integrity of the basal epidermal layer [1].

The diagnostic yield for EB ranges from 84% to 100%, with whole-exome sequencing (WES) providing a genetic diagnosis in 95% to 100% of cases across three patient cohorts [2]. In this study, we report seven genetic variants associated with EB, including four novel mutations, and demonstrate the pivotal role of WES in their detection.

## 2. Cases Presentation

Our cohort included patients with EB who had been under long-term clinical observation at the State Research Center of Dermatovenerology and Cosmetology. Genetic testing was carried out over a six-month period as part of ongoing diagnostic refinement and therapeutic planning. One newly referred patient (Case 7), who had not undergone long-term evaluation at the center, underwent testing to identify a second pathogenic variant in the *COL7A1* gene.

The following variants were identified in our cohort of patients with EB and were associated with their clinical phenotypes (Table 1).

### 2.1. Case 1

**Figure 1:**
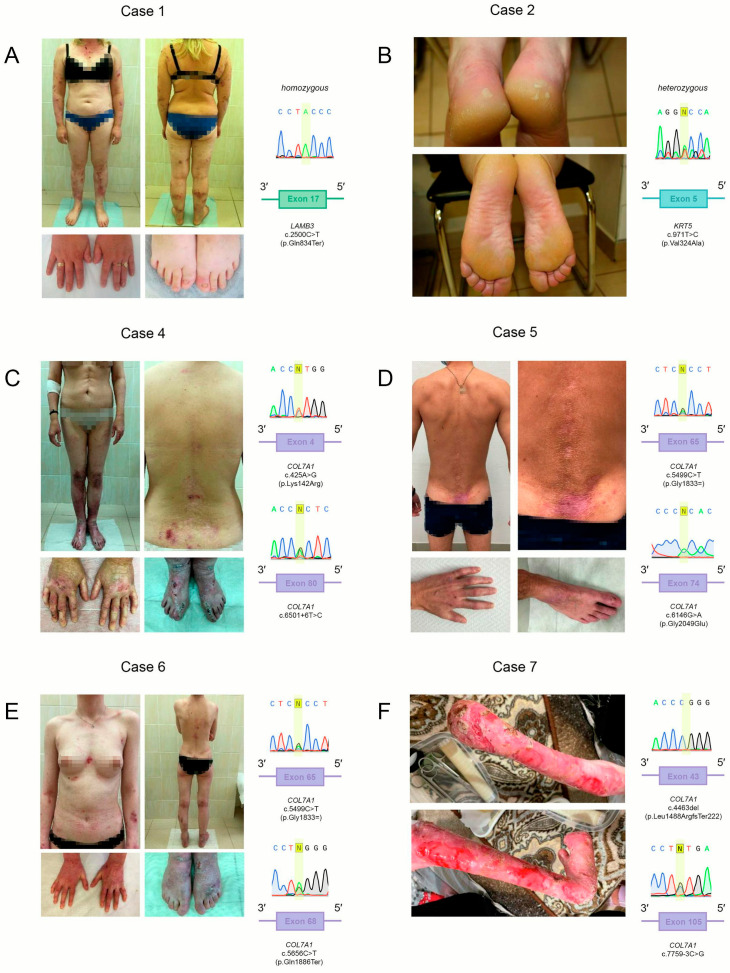
Clinical and morphological findings in patients. (**A**) Case 1: Junctional epidermolysis bullosa (EB). Clinical presentation (**left**) and Sanger sequencing showing the homozygous *LAMB3* variant (**right**). (**B**) Case 2: Simple generalized EB. Clinical presentation (**left**) and Sanger sequencing showing the heterozygous *KRT5* variant (**right**). (**C**–**F**) Cases 4–7: dystrophic EB. Clinical presentation (**left**) and Sanger sequencing of the *COL7A1* variants (**right**). Transcripts: *LAMB3*—NM_000228.3; *KRT5*—NM_000424.4; *COL7A1*—NM_000094.4.

A 33-year-old Caucasian female was admitted to the dermatology department of the State Research Center of Dermatovenerology and Cosmetology. Cutaneous involvement was limited to the limbs and manifested as localized thin scars, fragile blisters filled with clear fluid, and well-demarcated erosions with bright pink bases. Some areas contained serous, hemorrhagic, and purulent exudate. The patient reported intermittent severe pruritus, with no involvement of mucous membranes. Fingernails and toenails were partially preserved but appeared dystrophic, thickened, and discolored with a yellowish tint (Figure 1A, left). The total EBDASI score was 63, corresponding to a mild form of the disease [8].

The first clinical manifestations, characterized by skin blistering, were observed within two months after birth. At that time, the patient was diagnosed clinically with EB, without subtype classification or laboratory confirmation. Family history was unremarkable, with both parents showing no signs of EB. The patient’s mother had died prior to her admission to the clinic. The patient has one child, who is clinically unaffected.

The patient primarily relied on self-treatment, including the use of adhesive dressings (e.g., gauze bandages) and intermittent topical applications of dexpanthenol and Levomekol (dioxomethyltetrahydropyrimidine + chloramphenicol). During hospitalization, a human living skin equivalent was transplanted onto two non-infected wounds measuring 2.5 × 0.7 cm and 1 × 2 cm. By day 14 post-transplantation, clinical assessment revealed complete re-epithelialization of the treated wounds, with collagen VII (COLVII) expression remaining unchanged according to immunofluorescence antigen mapping. No new blisters or erosions developed at the graft sites by day 30. The patient received hydroxyzine 25 mg twice daily for pruritus and wound care using non-adhesive dressings (Branolind N, Mepitel, Mepilex Lite, and Licotul), changed daily for 14 days. Fusidic acid 2% cream was applied once daily to infected wounds. Upon discharge, the patient reported satisfaction with both clinical and laboratory outcomes.

Genetic analysis revealed a homozygous pathogenic single nucleotide variant (SNV) in exon 17 of the *LAMB3* gene (chr1:209623038G>A), resulting in a premature termination codon (PTC) at position Gln834 (p.Gln834Ter, NM_000228.3) in the β3 subunit of laminin 5 (Figure 1A, right). Both BayesDel addAF and BayesDel noAF in silico prediction tools classified the variant as pathogenic.

**Figure 2:**
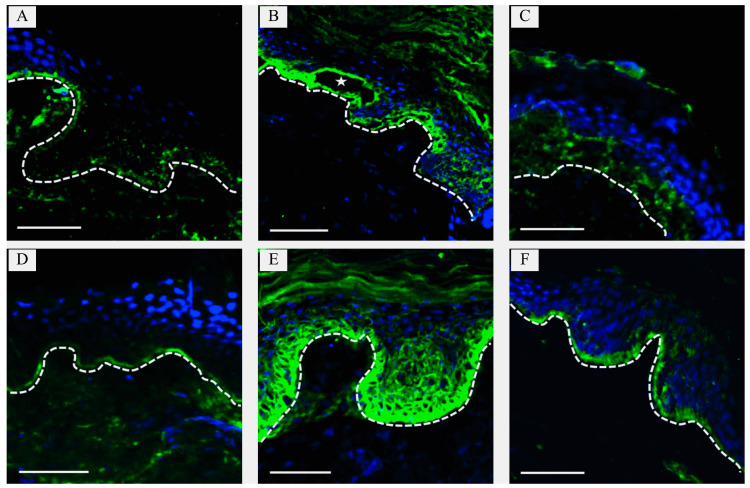
Immunohistochemical analysis of paraffin-embedded skin biopsy specimens. (**A**,**D**)—Laminin β3 staining using GTX103736. (**A**) Junctional epidermolysis bullosa (EB) (Case 1): discrete protein distribution. (**D**) Normal skin: continuous laminin β3 expression along the basement membrane. (**B**,**E**)—Keratin 5 staining using ab207351. (**B**) EB simplex (Case 3): asterisk denotes cleavage within the basal epidermal layer. (**E**) Normal skin: keratin 5 expression in both the basal and suprabasal layers of the stratified epidermis. (**C**,**F**)—Collagen VII staining using GTX89040. (**C**) Recessive dystrophic EB (Case 6): discrete and irregular collagen VII distribution. (**F**) Normal skin: uniform expression of collagen VII along the basement membrane. DAPI (blue) was used for nuclear counterstaining. Basement membrane is designated by a line. Scale bar: 100 µm.

Immunohistochemical (IHC) analysis of a skin biopsy stained for laminin β3 revealed focal positive staining, though the distribution was not specific to LAMβ3 within the basement membrane zone (Figure 2A,D).

### 2.2. Case 2 and Case 3

A 47-year-old male patient (Case 2) presented with localized eruptions on the torso, palms, and soles (Figure 1B, left). On the torso, the lesions appeared as erosions and crusts aligned with areas of mechanical friction from undergarments and waistbands. On the feet, residual epidermal tissue was visible at the periphery of healed erosions. Prominent plantar hyperkeratosis and hyperhidrosis were also observed, with patchy hyperkeratosis on the palms. The nail plates of the big toes showed mild deformities and transverse ridging. The total EBDASI score was 17, indicating a mild disease course.

Similar clinical features were noted in the patient’s 20-year-old Caucasian son (Case 3). His eruptions appeared on the soles, between the toes, and along the sides of the feet, characterized by blisters of varying sizes. The blisters were tense, with clear serous content; one blister exhibited hemorrhagic fluid. Additional findings included palmoplantar keratosis and marked plantar sweating. The patient’s total EBDASI score was 8.

The patients were hospitalized for clinical evaluation and treatment. In both cases, initial blistering appeared within the first month of life, affecting the soles. EB simplex (EBS) was diagnosed within the first year by a pediatrician, but neither IHC nor molecular testing was performed at that time. No other family members exhibited signs of EB. Dermatological follow-up was sporadic and limited to flare-ups. Self-management included the use of non-adhesive dressings and occasional topical agents such as dexpanthenol, methyluracil, and emollients. During hospitalization, wound care involved the application of non-adhesive dressings (Licotul and Polytul), changed daily or every other day for 14 days. Other interventions were limited to puncturing blisters with sterile needles. Both patients reported satisfaction with treatment outcomes and diagnostic clarification.

A skin biopsy from an affected area was obtained, and IHC staining for keratin 5 was performed, which illustrates cleavage at the basal epidermis (Figure 2B), consistent with intraepidermal blistering. For comparison, Figure 2E displays keratin 5 distribution in morphologically normal skin from a healthy control.

WES of peripheral blood DNA revealed a heterozygous rs59335325 variant in exon 5 of the *KRT5* gene (chr12:52517711A>G), resulting in a Val324Ala substitution (NM_000424.4) (Figure 1B, right). In silico pathogenicity meta-predictors classified this variant as pathogenic (BayesDel addAF, BayesDel noAF, MetaRNN), while others (MetaLR, MetaSVM, REVEL) designated it as a variant of uncertain clinical significance.

### 2.3. Case 4

A 45-year-old Caucasian female presented with predominant erosive lesions of the esophageal mucosa and dysphagia. Physical examination revealed extensive areas of atrophic scarring involving the back, hands, and extensor surfaces of the elbows and knees (Figure 1C, left). Small erosions (<1 cm in diameter) were observed without clinical signs of secondary infection. Notable complications included microstomia, ankyloglossia, fusion of the vestibular oral folds, congenital anonychia, and esophageal stenosis. The patient’s clinical condition was assessed with an EBDASI score of 124, corresponding to severe disease.

The patient developed plantar blistering at birth, which subsequently spread to other anatomical sites. At one year of age, she was diagnosed with dystrophic EB (DEB), confirmed histologically from a skin biopsy. At that time, IHC and genetic investigations were not available. Family history was non-contributory. The patient has one child, who is clinically unaffected. Throughout her life, the patient has rarely sought dermatological care. For symptomatic management, she uses sea buckthorn oil to alleviate pain related to esophageal erosions and occasionally applies methyluracil ointment and emollients for skin lesions. Notably, she does not routinely use wound dressings.

During hospitalization at the State Research Center of Dermatovenerology and Cosmetology, the patient received symptomatic treatment, including the application of non-adhesive dressings (Mepilex, Mepilex Lite, Branolind N). Dressings were changed every two days over a 14-day period. Clinical reassessment demonstrated re-epithelialization of 90% of the erosive skin lesions.

A lesional skin biopsy was obtained and subjected to immunofluorescence analysis. The results demonstrated reduced expression of COLVII at the dermal–epidermal junction, thereby confirming the clinical diagnosis of DEB.

A recurrent nucleotide sequence variant in exon 4 of the *COL7A1* gene, c.425A>G (chr3:48593538T>C), was identified in a heterozygous state (Figure 1C, right top), leading to an amino acid substitution at position 142 of the protein (p.Lys142Arg, NM_000094.4). This alteration has been reported to disrupt RNA splicing by impairing the downstream donor splice site (DSS) [5].

No additional pathogenic variants were detected in the coding regions of *COL7A1*. However, a heterozygous intronic variant, c.6501+6T>C (chr3:48574256A>G, intron 80), was identified (Figure 1C, right bottom).

### 2.4. Case 5

A 42-year-old Caucasian male was referred for dermatological evaluation. Clinical examination revealed multiple areas of atrophic scarring involving the lower back and inguinal folds (Figure 1D, left), in addition to active skin wounds and crusting. Isolated erosions, each measuring up to 1 cm in diameter, were identified within the inguinal folds. Partial congenital anonychia was observed, although several nail plates remained intact on both hands and feet. The patient also exhibited microstomia, ankyloglossia, and fusion of the vestibular folds within the oropharyngeal region. The total EBDASI score was 46, corresponding to moderate disease.

The patient presented at birth with palmoplantar blistering and congenital anonychia of the feet. Within the first few months of life, esophageal involvement became clinically apparent. A clinical diagnosis of DEB was established during early childhood; however, histological analysis was not performed. Following the transition to adulthood, the patient remained outside of specialized medical care for an extended period and managed the disease independently. He did not utilize wound dressings, instead applying dexpanthenol cream and a topical enzymatic preparation containing clostridiopeptidase and protease during periods of disease exacerbation.

Upon admission to the State Research Center of Dermatovenerology and Cosmetology, the patient was managed with non-adherent dressings (Licotul, Polytul). Dressings were changed every two days for 14 days. Complete re-epithelialization of erosive lesions was documented at follow-up.

To confirm the clinical diagnosis, IHC staining of a lesional skin biopsy was performed. Immunofluorescence revealed markedly reduced expression of COLVII at the dermal–epidermal junction, consistent with DEB.

The patient was found to carry compound heterozygous variants in the *COL7A1* gene. A previously described heterozygous nucleotide substitution in exon 65 (chr3:48578354G>A) was detected (Figure 1D, right top) [6]. In silico analyses predict this variant to be benign, supported by a low evolutionary conservation score (PhyloP100way = –0.393).

In addition, a recurrent heterozygous variant was identified in exon 74 (chr3:48575373C>T), resulting in a missense substitution p.Gly2049Glu (NM_000094.4) (Figure 1D, right bottom) [7]. This alteration resides within a known 17-amino-acid pathogenic hotspot. All ensemble in silico pathogenicity meta-predictors (BayesDel addAF, BayesDel noAF, MetaLR, MetaRNN, MetaSVM, REVEL) consistently classified this variant as pathogenic.

### 2.5. Case 6

A 23-year-old Caucasian female was referred to the dermatology department of the State Research Center of Dermatovenerology and Cosmetology. Physical examination revealed a flaccid blister approximately 4 cm in diameter with a smooth contour, located on the medial aspect of the left shin. On the flexor surface of the left knee, an irregularly shaped erosion with well-defined margins and residual epidermal fragments was observed (Figure 1E, left). Re-epithelializing erosions with surrounding erythema and epidermal remnants, measuring 2 × 3 cm, were present on the extensor surfaces of the elbows. Additional findings included anonychia, microstomia, a shortened lingual frenulum, and two erosive lesions (up to 1 cm in diameter) on the dorsal surface of the tongue. The oral cavity was otherwise unremarkable. The total EBDASI score was 106, corresponding to moderate disease severity. A skin biopsy was not performed.

Initial manifestations were noted at birth in the form of plantar blistering. At the age of 3, a clinical diagnosis of EB was performed, but histological analysis was not performed. At home, the patient used dexpanthenol-based topical agents and emollients, with only occasional use of adherent dressings.

In the hospital setting, a human living skin equivalent was transplanted onto two non-infected wounds measuring 2 × 0.8 cm and 1 × 0.4 cm. By day 14 post-transplantation, complete wound closure was observed. By day 30, no new blisters or erosions were detected at the graft site.

IHC analysis revealed disrupted distribution of COLVII at the dermo-epidermal junction compared with normal skin, supporting the diagnosis of a COLVII-associated disorder (Figure 2C,F).

A previously undescribed nucleotide sequence variant in exon 68 of the *COL7A1* gene (chr3:48576720G>A) was identified in a heterozygous state, creating a PTC at position 1886 (p.Gln1886Ter, c.5656C>T, NM_000094.4) (Figure 1E, right top). This type of nucleotide sequence variant is a known cause of the disease. According to meta-prediction algorithms (BayesDel addAF, BayesDel noAF), this variant is classified as pathogenic. In addition, the patient carried the synonymous variant *COL7A1* p.Gly1833= in a heterozygous state.

Given the presence of a PTC, which may be amenable to read-through therapy [9], the patient was administered 0.1% gentamicin sulfate ointment topically three times daily for two weeks. By day 4 of treatment, complete epithelialization of a 1 cm erosion was achieved.

### 2.6. Case 7

The patient’s cutaneous manifestations were widespread, predominantly affecting the forearms and hands. Large hemorrhagic erosions were present on the left forearm and hand, some covered by serous and hemorrhagic crusts. Deformity and flexion contracture of the left wrist were noted, along with absent nails on the left hand (Figure 1F, left). Pseudosyndactyly and contractures of digits 2–5 were also observed on the left hand. The patient had bilateral hand contractures since age 3, with marked involvement of the right fifth digit. Surgical intervention for syndactyly was performed to restore digital separation, preserving mobility in the interphalangeal joints.

At age 11, the skin over the thighs remained unaffected, and contractures on the hands were surgically excised. Over time, most wounds did not heal fully but fluctuated in size, with seasonal worsening in spring and summer. At age 18, the patient was diagnosed with squamous cell carcinoma (SCC) of the lower limb, necessitating surgical excision. Further investigations, including ultrasound, computed tomography (CT), and lymph node biopsy, were performed. At age 19, additional SCC lesions and axillary lymph node metastases were excised. The patient remains under continuous oncologic surveillance with regular CT imaging. The total EBDASI score was 162, consistent with severe disease.

A novel frameshift variant in exon 43 of the *COL7A1* gene (chr3:48583145CA>C) was identified in a heterozygous state (p.Leu1488ArgfsTer222, NM_000094.4), known to be pathogenic (Figure 1F, right top).

Additionally, a previously unreported SNV was identified in intron 104 of the *COL7A1* gene (chr3:48568537G>C) in a heterozygous state (c.7759-3C>G, NM_000094.4) (Figure 1F, right low). The dbscSNV algorithm predicted this variant disrupted splicing (score = 0.999827). This variant was also found in a heterozygous state in the patient’s clinically unaffected mother.

## 3. Discussion

Our findings suggest that, for the cases under consideration, the type of mutation may have a more significant impact on disease severity in recessive DEB (RDEB) than its domain localization, although this observation is derived from a limited number of cases and should be interpreted with caution. This is exemplified by Case 4, the only patient with a variant located exclusively in the NC1 domain on one allele, who exhibited severe systemic manifestations. While this case also harbors a second variant in the THD domain on the other allele, the lack of biallelic THD involvement contrasts with Gupta et al.’s report associating THD variants with pronounced systemic involvement [10]. Importantly, while Gupta et al.’s cohort primarily comprised pediatric patients (median age 15 months; only 17 individuals >10 years), our study focused on adults (>18 years), providing valuable insights into long-term disease progression and phenotypic variability in this population. Nevertheless, the small sample size and age difference between cohorts preclude definitive conclusions.

### 3.1. Case 1

The *LAMB3* gene encodes the β3 subunit of laminin-332, which is essential for the structural integrity of the basement membrane and anchorage of epithelial cells. Pathogenic variants in *LAMB3* are a known cause of junctional ED (JEB), predominantly its lethal subtype, although non-lethal forms have also been described [11,12]. Clinical outcomes vary depending on the type of variant: nonsense mutations generally result in severe phenotypes, while some in-frame skipping events may produce milder disease [3,13]. Common pathogenic variants include R635X and R42X, with specific mutations correlating with varying disease severities, emphasizing the importance of nucleotide context for mRNA stability and laminin-332 production.

In this case, we report a patient harboring a homozygous p.Gln834Ter variant (rs770868939), previously identified only in compound heterozygous states in individuals with mild JEB. This nonsense mutation likely reduces splicing enhancer activity, leading to exon 17 skipping and the production of a truncated but partially functional β3 subunit of laminin-332 [3]. The rs770868939 variant was found in a heterozygous state in one individual of European ancestry within the gromAD control cohort and was also discovered in the RUSeq database among healthy individuals/patients with a frequency of 0/0.000104 (one affected).

### 3.2. Case 2 and Case 3

Heterozygous pathogenic variants in *keratin 5* (*KRT5*) or *keratin 14* (*KRT14*) account for up to 75% of cases of EBS, the most prevalent form of EB. In Case 3, a missense variant was identified in KRT5 at amino acid residue 324, located within the L12 non-helical linker segment of the keratin 5 protein. Other variants reported in this region (e.g., positions 327, D328E, and 329) are not typically associated with severe phenotypes or keratin filament clumping but are known to interfere with the obligate heterodimerization between KRT5 and KRT14—an interaction critical for keratin network stability.

The p.Val324Leu variant has not been reported in the gnomAD or RUSeq databases and was identified in a patient diagnosed with the Weber–Cockayne type of EBS (EBS-WC), characterized by blistering localized to acral friction sites [4]. Notably, this was the first reported case of amyloid deposition in the papillary dermis in a patient with EBS-WC. The same variant was also detected in a 61-year-old individual with localized EBS type 2C and secondary cutaneous amyloidosis (onset in childhood), as well as in a 19-year-old female with localized EBS type 2C since birth [14].

### 3.3. Case 4

The *COL7A1* variant c.425A>G (p.Lys142Arg) is a well-documented pathogenic mutation in DEB, reported in both homozygous and compound heterozygous states. In the European (non-Finnish) population, its allele frequency is 0.000095 in gnomAD. In previous studies, this variant was found in a heterozygous state in 1 out of 100 control alleles, indicating its low frequency among healthy individuals. The p.Lys142Arg variant has notable prevalence in European populations of RDEB, ranging from localized to generalized and severe forms [5,15,16]. This variant has been detected on 34/268 (9.5%) alleles in 27 (16%) unrelated families among Russian patients with RDEB and was described as the most common mutation in the *COL7A1* gene [17,18]. Aberrant splicing results in the formation of two alternative shortened transcripts containing PTCs, which undergo nonsense-mediated mRNA decay (NMD), resulting in a deficiency of the protein product [5].

In earlier studies, we demonstrated the presence of the correctly spliced transcript containing the homozygous c.425A>G variant in patient cells. However, alongside the correct transcript, we also identified aberrant transcripts resulting from faulty splicing. Although we successfully sequenced the transcript encoding the p.Lys142Arg variant within the open reading frame of *COL7A1*, the splicing efficiency was found to be low [17]. For this pathogenic variant, gene therapy using CAS9n-mediated genome editing has shown promising results, although these findings have been demonstrated only in patient-derived cell lines [19]. This therapeutic approach may hold potential for future treatments of RDEB patients.

Additionally, the patient carried a second variant in intron 80 of *COL7A1* (chr3:48574256A>G) (c.6501+6T>C, NM_000094.4) (Figure 1C, right low), likely impacting DSS formation as predicted by the dbscSNV (score = 0.7168) (splicing disruption due to this variant is discussed in 3.7). This variant is absent in both gnomAD and RUSeq.

### 3.4. Case 5

A heterozygous variant of *COL7A1* (NM_000094.4):c.5499C>T (p.Gly1833=) was identified. Although synonymous, this variant has previously been reported in a homozygous state in a patient with RDEB [6]. It caused aberrant splicing of exon 64 and a resulting shortened transcript due to skipping the last 35 nucleotides of exon 64. Also, it was identified as part of a compound heterozygous genotype with p.Gly2028Argfs*71 in a Chinese patient presenting with congenital RDEB [20]. This synonymous mutation p.Gly1833= was also noted in a targeted next-generation sequencing (NGS) study of Russian pediatric patients with genodermatoses, where it appeared in 10 alleles [18]. Its frequency in the gnomAD control cohort is 0.00002418, with no homozygotes reported. In the RUSeq database, it was found in both healthy individuals and patients at frequencies of 0.0002966 and 0.0001043, respectively (one healthy, one affected individual).

The missense variant p.Gly2049Glu, though not registered in gnomAD or RUSeq, has been previously reported in patients with diverse RDEB phenotypes, including inversa, generalized intermediate, and generalized severe forms [7].

### 3.5. Case 6

We identified the *COL7A1* variant (NM_000094.4):c.5656C>T (p.Gln1886Ter) resulting in a PTC in the open reading frame of the gene in the patient from Case 6 and administered topical gentamicin. As members of the aminoglycoside class, agents like gentamicin are employed in treating various forms of EB, especially RDEB, to restore functional protein expression in patients harboring PTC mutations [21]. Importantly, these compounds facilitate PTC read-through without significantly causing misreading at natural stop codons in eukaryotic cells [22]. The efficacy and safety of gentamicin read-through therapy for RDEB patients with nonsense mutations have been widely studied and discussed [22,23]. Systemic administration is considered the most effective delivery method as it enables gentamicin to reach otherwise inaccessible tissues, including the epithelial and mucous membranes of internal organs. In a recent open-label pilot trial (2018–2020), two intravenous gentamicin regimens were tested, with follow-up extending to 180 days post-treatment [9]. Topical gentamicin application could stimulate anchoring fibril formation at the dermal–epidermal junction without adverse side effects [24]. Recently, a new aminoglycoside analog, ELX-02, has been developed specifically to enhance PTC read-through. Studies indicate that ELX-02 exhibits greater efficacy than gentamicin in EB models [25].

The p.Gln1886Ter variant identified in our study was not documented in the gnomAD control dataset and is absent from the RUSeq database.

Despite being nearly two decades younger than Case 5, Case 6 exhibited a more severe clinical phenotype (EBDASI 106 in Case 6 and EBDASI 46 in Case 5). The nonsense mutation (p.Gln1886Ter) in Case 6 likely caused greater protein dysfunction than the missense variant (p.Gly2049Glu) in Case 5. While gentamicin promoted lesion healing in Case 6, the overall disease burden remained higher, consistent with the more disruptive nature of truncating mutations.

### 3.6. Case 7

**Figure 3:**
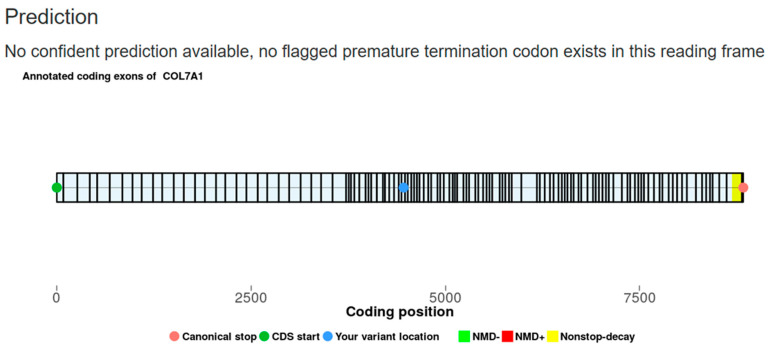
Prediction of nonsense-mediated mRNA decay (NMD) escape for the frameshift variant c.4463del (p.Leu1488ArgfsTer222, NM_000094.4) identified in Case 7. The prediction was performed using the NMDEscPredictor algorithm [27]. No premature termination codons were flagged in the open reading frame, although the prediction does not confidently confirm NMD escape for the aberrant transcript.

This patient harbored a heterozygous SNV in exon 43 of *COL7A1* (chr3:48583145CA>C), leading to a frameshift mutation (p.Leu1488ArgfsTer222, NM_000094.4). This variant (rs2044901784) was found in a heterozygous state in one individual from Finland in the gnomAD control dataset but was not registered in RUSeq. While DSS mutations (e.g., c.4482+1G>A) affecting exon 43 have been associated with DEB [15], this frameshift variant results in a novel open reading frame extending across 222 codons, with a PTC located in exon 58. Because the PTC is situated only 26 bp downstream of the nearest exon–exon junction, it does not meet the 50-nucleotide threshold required to trigger NMD [26,27]. Consequently, this transcript is likely to escape NMD, generating a truncated protein. Interestingly, NMD-escape transcripts may possess gain-of-function properties or exert dominant-negative effects, even though no evidence of toxic protein accumulation has been observed in this case. This variant has been confirmed as recessive as the same frameshift mutation was identified in the patient’s healthy father. The NMD escape intolerance prediction in silico tool (Figure 3) did not flag any PTCs in the open reading frame [28]. Although confident predictions are unavailable, this does not rule out the possibility that the altered transcript may still impact cellular homeostasis.

In search of a second variant contributing to the severe RDEB phenotype in this patient, we identified an intronic sequence variant in another *COL7A1* allele (c.7759-3C>G, NM_000094.4), which has not been previously described. The variant frequency in the gnomAD control dataset is 0.000002489 (three heterozygotes), and it was not detected in RUSeq. We registered it in the Leiden Open Variation Database (LOVD): https://databases.lovd.nl/shared/variants/0000987839#00000019 (accessed on 9 November 2024). The putative role of splicing disruption due to this variant is discussed in Section 3.7.

Both *COL7A1* variants in Case 7 are novel and lead to severe RDEB, potentially contributing to disease severity by altering protein expression level or functionality. Even in the absence of predicted NMD escape, the aberrant transcript may still produce a dysfunctional protein, exacerbating clinical severity.

### 3.7. Intronic Sequence Variants of COL7A1 Associated with Aberrant Splicing

The *COL7A1* variant disrupts a purine-rich splicing enhancer region, potentially inducing natural exon skipping that bypasses PTCs and restores the reading frame [29]. Notably, all exons containing novel loss-of-function (LOF) variants were in-frame, making them theoretically skippable. Our findings establish the pathogenicity of non-canonical splice site variants in *COL7A1*, underscoring the critical need to evaluate intronic variants in NGS analyses. Although whole-exome sequencing generally lacks coverage of intronic regions, *COL7A1* represents an exception due to its characteristically short introns.

**Figure 4:**
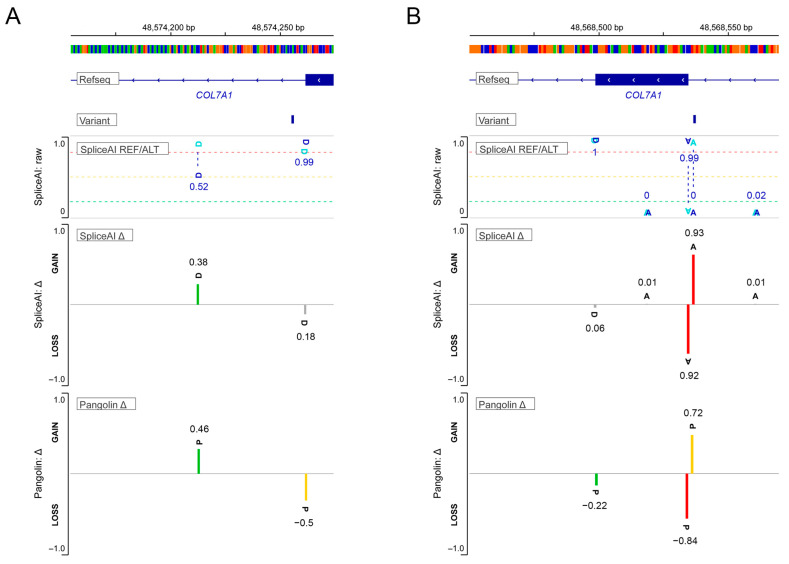
Schematic representations illustrating SpliceAI predictions for the newly identified variants in Case 4 (c.6501+6T>C) and Case 7 (c.7759-3C>G). (**A**) The c.6501+6T>C variant (Case 4) is predicted to create a novel donor splice site downstream, presumably resulting in the inclusion of intron 80. (**B**) The c.7759-3C>G variant (Case 7) is expected to significantly affect the acceptor splice site, likely leading to the deletion of exon 105 (https://spliceailookup.broadinstitute.org/ (accessed on 10 September 2024)). *COL7A1*—NM_000094.4. Delta scores indicate the predicted likelihood of splicing impact: red (≥0.8) denotes high confidence, yellow (≥0.5) likely impact, green (≥0.2) possible impact, and gray (<0.2) little or no predicted effect.

To assess the potential impact of the newly identified variants on splicing, we analyzed them using two computational prediction tools: SpliceAI and Pangolin. Both tools predicted that these variants could cause splicing abnormalities (Figure 4). For the intronic variant in *COL7A1* (chr3:48574256A>G; c.6501+6T>C, NM_000094.4) identified in Case 4, the dbscSNV algorithm predicted a 0.7168 probability of DSS disruption. SpliceAI analysis further indicated the highest probability of a donor gain event (Δ score = 0.38) occurring 43 bp downstream of the variant position (Figure 4A).

Although this variant does not directly affect the canonical ±1–2 splice site nucleotides, the +6 position lies within the consensus sequence recognized by the splicing machinery. Potential consequences include reduced splicing efficiency, intron retention, or incorporation of additional intronic sequences, any of which could render type VII collagen nonfunctional. Regarding nearby variants, c.6501+1G>C has been established as pathogenic in EB [30], while c.6501+7G>A is classified as probably benign in ClinVar. In contrast, c.6501+8G>T carries a variant of uncertain significance (VUS) designation from a single submitter, with reported association with RDEB. The c.6500-1G>C variant, documented in RDEB cases (ClinVar: VCV000106770.1), induces either exon skipping or cryptic splice site activation.

In silico analysis of the novel intronic variant in *COL7A1* (chr3:48568537G>C; c.7759-3C>G, NM_000094.4; Case 7) suggested potential splice site disruption. Supporting this prediction (Figure 4B), SpliceAI scores indicated a high probability of acceptor site alteration, with a 0.92 loss score for the canonical −3 position and a 0.93 gain score for a new acceptor site 1 bp upstream. These findings were corroborated by Pangolin, which predicted a 0.85 splice loss at −3 bp and a 0.72 gain at −1 bp upstream. However, the proximity of this alternative acceptor site (just 2 bp from the original) is insufficient to maintain normal splicing, instead favoring exon 105 skipping. This splicing defect would likely produce dysfunctional type VII collagen.

Several intronic mutations in the *COL7A1* gene, such as c.6500-1G>C, c.5818+5G>A, c.6636-1G>A, and c.6657+1G>A, disrupt normal splicing by influencing splice donor or acceptor sites, from partial impairment to complete loss. These variants frequently occur near splice sites, typically within the –3 to +3 region, leading to outcomes like exon skipping, frameshifts, or the use of cryptic splice sites. This may result in in-frame deletions or PTCs, compromising protein function and stability [31].

Variants like c.6750+2T>G and c.6869+1G>A disrupt DSS, causing abnormal splicing, while variants such as c.7799+1G>A and c.7759-3C>G affect acceptor sites, contributing to RDEB pathogenesis through impairing COLVII production. These findings are corroborated by several databases, including ClinVar ([VCV000010055] (https://www.ncbi.nlm.nih.gov/clinvar/variation/VCV000010055/ (accessed on 9 November 2024))) and HGMD ([CM033304] (http://www.hgmd.cf.ac.uk/ac/index.php (accessed on 9 November 2024))), providing crucial insights into how splicing mutations drive disease progression in DEB (https://databases.lovd.nl/shared/view/COL7A1 (accessed on 9 November 2024), https://www.ncbi.nlm.nih.gov/clinvar/variation/29636/ (accessed on 9 November 2024)). A recent study identified a deep intronic variant in *COL7A1* (c.7795-97C>G) that induces pseudoexon insertion between exons 104 and 105 [32].

Given that splicing mutations frequently cause the most severe disruptions to gene expression, developing targeted therapeutic strategies remains a critical priority. Among emerging approaches, modified U1 snRNAs show particular promise, having successfully restored canonical splicing for 5′ splice site mutations at +1/+2 positions [33]. This proof of concept demonstrates the potential of RNA-targeted interventions to address these challenging genetic defects.

For example, antisense oligonucleotides (ASOs) can modulate splicing by blocking interactions between intronic splicing silencers (ISSs) or exonic splicing enhancers (ESEs) and the spliceosome, resulting in either exon skipping or inclusion. This approach enables the generation of alternatively spliced transcripts that exclude pathogenic or toxic sequences. ASOs designed to target exon–intron junctions can alter the balance between alternative splice isoforms or induce complete exon skipping. Notably, several ASOs targeting genes involved in hereditary disorders caused by aberrant splicing have recently gained FDA approval [34,35,36]. Bornert et al. demonstrated the therapeutic potential of ASOs by successfully modulating *COL7A1* exon 73 splicing in both RDEB and DDEB forms [37]. A double-blind, placebo-controlled clinical trial (NCT05529134) is currently underway to evaluate the safety, mechanism of action, preliminary efficacy, and systemic exposure of PTW-002 in DEB patients with *COL7A1* exon 73 mutations.

Exons 43, 68, 80, and 105 reside within the triple-helix domain and exclusively encode Gly-X-Y repeat sequences. Notably, work by Turczynski et al. targeting exons 73 and 80 of COL7A1 demonstrated their dispensability for type VII collagen function [35]. In their study, ASO-mediated exon skipping in primary cells restored up to 36% of wild-type COLVII expression. Subsequent subcutaneous administration in a mouse model yielded functional recovery, with detectable collagen expression and anchoring fibril formation.

Type VII collagen lacking the amino acids encoded by exon 105 (81 bp) retained normal functionality in biochemical and cellular assays, including triple-helix thermostability, fibroblast migration, and adhesion. Upon injection into a mouse model, this modified collagen properly incorporated into the basement membrane zone. These findings indicate that exon 105 skipping does not compromise protein function [38]. ASO-mediated exon 105 skipping successfully restored functional type VII collagen production in vitro, using both keratinocytes and fibroblasts, and achieved therapeutic expression levels in vivo in a mouse model. These results provide a compelling proof of concept for antisense oligonucleotide (AON)-mediated exon skipping as a potential systemic therapy for RDEB [36].

In the study by Bremer J. et al., the correlation between *COL7A1* expression levels and disease severity in RDEB was investigated [39]. Specifically, patients with acral, pretibial, and generalized intermediate forms of RDEB exhibited normal expression levels, while those with more severe forms showed reduced or even complete absence of COLVII. Surprisingly, patients with generalized severe RDEB—who were expected to benefit from exon-skipping mutations that preserve protein production—showed a complete absence of COLVII. This unexpected finding suggests alternative pathogenic mechanisms, such as low-level exon 107 skipping or the presence of concurrent null mutations. Our results further highlight the potential of COLVII expression as a biomarker for assessing RDEB severity and monitoring treatment response.

A case of a 60-year-old Japanese woman with persistent blistering and scarring on the pretibial region and elbows was reported in the context of pretibial RDEB. Mutational analysis revealed a c.5797C>T mutation in exon 70 (p.R1933X) and a c.6348+1G>A mutation in intron 76 of *COL7A1*. Reverse transcription polymerase chain reaction demonstrated that the c.6348+1G>A mutation led to exon 76 skipping and intron 76 retention, with both transcripts being in-frame [40]. These findings suggest that intron retention during exon skipping—particularly in regions encoding the triple-helix domain when occurring in compound heterozygosity with LOF variants—may represent a novel pathogenic mechanism underlying RDEB.

## 4. Materials and Methods

### 4.1. WES

Libraries were prepared from 500 ng of genomic DNA using the MGIEasy Universal DNA Library Prep Set (MGI Tech, Shenzhen, China), following the manufacturer’s protocol. DNA fragmentation was performed using ultrasound on a Covaris S-220 device, targeting an average fragment length of 250 bp, and we utilized SureSelect Human All Exon v7 and v8 probes (Agilent Technologies, Santa Clara, CA, USA), which cover the entire human exome [41]. DNA and libraries concentrations were measured using a Qubit Flex (Life Technologies, Carlsbad, CA, USA) with a dsDNA HS Assay Kit (Invitrogen, Waltham, MA, USA), adhering to the manufacturer’s protocol. The quality of the libraries was assessed on a Bioanalyzer 2100 instrument with the High Sensitivity DNA kit (Agilent Technologies, Santa Clara, CA, USA), also in accordance with the manufacturer’s guidelines. The libraries were circularized and sequenced in a paired-end mode on the platform DNBSEQ-G400 by using DNBSEQ-G400RS High-throughput Sequencing Set PE100 (MGI Tech, Shenzhen, China), achieving an average coverage of 100×.

FastQ files were generated using the basecallLite software (ver. 1.0.7.84) from the manufacturer (MGI Tech, Shenzhen, China). Bioinformatics quality control of the obtained sequencing data was performed using FastQC v0.11.9 (https://www.bioinformatics.babraham.ac.uk/projects/fastqc/ (accessed on 19 January 2024)). Based on the quality control, raw reads were corrected using bbduk v38.96 (https://sourceforge.net/projects/bbmap/ (accessed on 19 January 2024)).

Next reads of each sample were aligned to human genome (GRCh38) with bwa-mem2 v2.2.1 (https://github.com/bwa-mem2/bwa-mem2 (accessed on 19 January 2024)) and SAMtools v1.9 (https://github.com/samtools/samtools (accessed on 19 January 2024)), obtaining quality metrics for exome enrichment using Picard software v2.22.4 (https://broadinstitute.github.io/picard/ (accessed on 19 January 2024)), variant calling with bcftools v1.9 (https://github.com/samtools/bcftools (accessed on 20 January 2024)) and DeepVariant v1.5.0 (https://github.com/google/deepvariant accessed on 20 January 2024), and variant annotation using AnnoVar (https://annovar.openbioinformatics.org/en/latest/ (accessed on 20 January 2024)), InterVar v2.2.2 (https://github.com/WGLab/InterVar (accessed on 20 January 2024)), along with a number of custom scripts to optimize and improve the quality of the content in the final variant annotation files. MultiQC software v1.16 (https://multiqc.info/ (accessed on 21 January 2024)) was launched as a concluding quality control step. The interpretation of the clinical significance of the identified variants was carried out according to ACMG criteria [42] using variant databases and literature sources. All found variants were confirmed by Sanger sequencing.

### 4.2. IHC

Excisional biopsy was performed for immunofluorescence antigen mapping. Biopsy specimens were sampled within perilesional clinically normal-looking skin sites.

Skin samples were embedded in a special compound (Surgipath, Richmond, IL, USA; FSC 22 Clear) and frozen. Cryo-blocks were sectioned on a cryo-microtome, washed with phosphate-buffered saline (PBS) to be cleaned from cryo-compound, and then incubated with BSA-solution. The primary antibodies (1:200 anti-LAMβ3 (GeneTex, Irvine, CA, USA; GTX103736), 1:500 anti-cytokeratin-5 (Epitomics, Burlingame, CA, USA; AC-0027) and 1:100 anti-COL7A1 (GeneTex, Irvine, CA, USA; GTX37733)), were diluted with the BSA solution and incubated overnight at 4 °C. The slices were rinsed with PBS, followed by an incubation at 37 °C with Alexa Fluor 488-conjugated secondary antibodies (1:500 anti-rabbit IgG (GeneTex, Irvine, CA, USA; GTX213110-04)). Finally, the slices were mounted with Fluoroshield containing DAPI medium (GeneTex, Irvine, CA, USA; GTX30920) to visualize the nuclei. Fluorescence images were captured using an Olympus IX81S1F-S confocal laser scanning microscope (Olympus, Hachioji, Tokyo).

## 5. Conclusions

Our study presents novel findings that advance the genetic and clinical understanding of EB. In a small cohort of EB patients, we identified clinically significant variants in the *LAMB3*, *KRT5*, and *COL7A1* genes, including four previously unreported mutations. Notably, we describe two novel pathogenic variants in *COL7A1*: a frameshift mutation (p.Leu1488ArgfsTer222), predicted to escape NMD, and an intronic mutation (c.7759-3C>G) with strong in silico evidence of splice site disruption. Both variants were associated with severe RDEB phenotypes, highlighting the clinical and genetic heterogeneity of EB. These findings underscore the importance of detecting pathogenic intronic variants through whole-exome sequencing, particularly given the short introns of *COL7A1*, which may obscure their pathogenic potential.

Additionally, we report the successful application of gentamicin read-through therapy in an adult RDEB patient harboring a nonsense *COL7A1* mutation (p.Gln1886Ter), supporting its therapeutic promise. Our exclusively adult cohort provided unique insights into long-term phenotypic variability, revealing striking differences in disease severity among patients sharing one pathogenic *COL7A1* variant but differing in their second allele. This observation emphasizes the influence of mutation type and allelic context on clinical outcomes. Collectively, our findings advocate for integrating comprehensive genomic and functional analyses into EB diagnostics and management.

## Figures and Tables

**Table 1:** The variants of the small cohort of epidermolysis bullosa patients.

Gene	cDNA Change	Protein Change	rs ID	ACMG Classification (According to VarSome)	ACMG Criteria	Previously Described
*LAMB3* (NM_000228.3)	c.2500C>T	p.Gln834Ter	rs770868939	LP	PVS1, PM2	[3]
*KRT5* (NM_000424.4)	c.971T>C	p.Val324Ala	rs59335325	P	PM1, PM2, PM5, PP3	[4]
*COL7A1* (NM_000094.4)	c.425A>G	p.Lys142Arg (aberrant splicing)	rs121912856	P	PM2, PP3, PP5	[5]
	c.4463del	p.Leu1488ArgfsTer222	rs2044901784	P	PVS1, PM2, PP5	No
	c.5499C>T	p.Gly1833= (aberrant splicing)	rs758886532	LP	PM2, PP5, BP4	[6]
	c.5656C>T	p.Gln1886Ter	rs2044329211	P	PVS1, PM2, PP5	No
	c.6146G>A	p.Gly2049Glu	rs1410793870	P	PM1, PM2, PM5, PP3, PP5	[7]
	c.6501+6T>C		rs752070397	VUS	PM2	No
	c.7759-3C>G		rs1030248456	LP	PM2, PP3, PP5	No

Notes: P: pathogenic; LP: likely pathogenic; VUS: variant of uncertain significance.

## Data Availability

The data that support the findings of this study are available from the corresponding author upon reasonable request.
